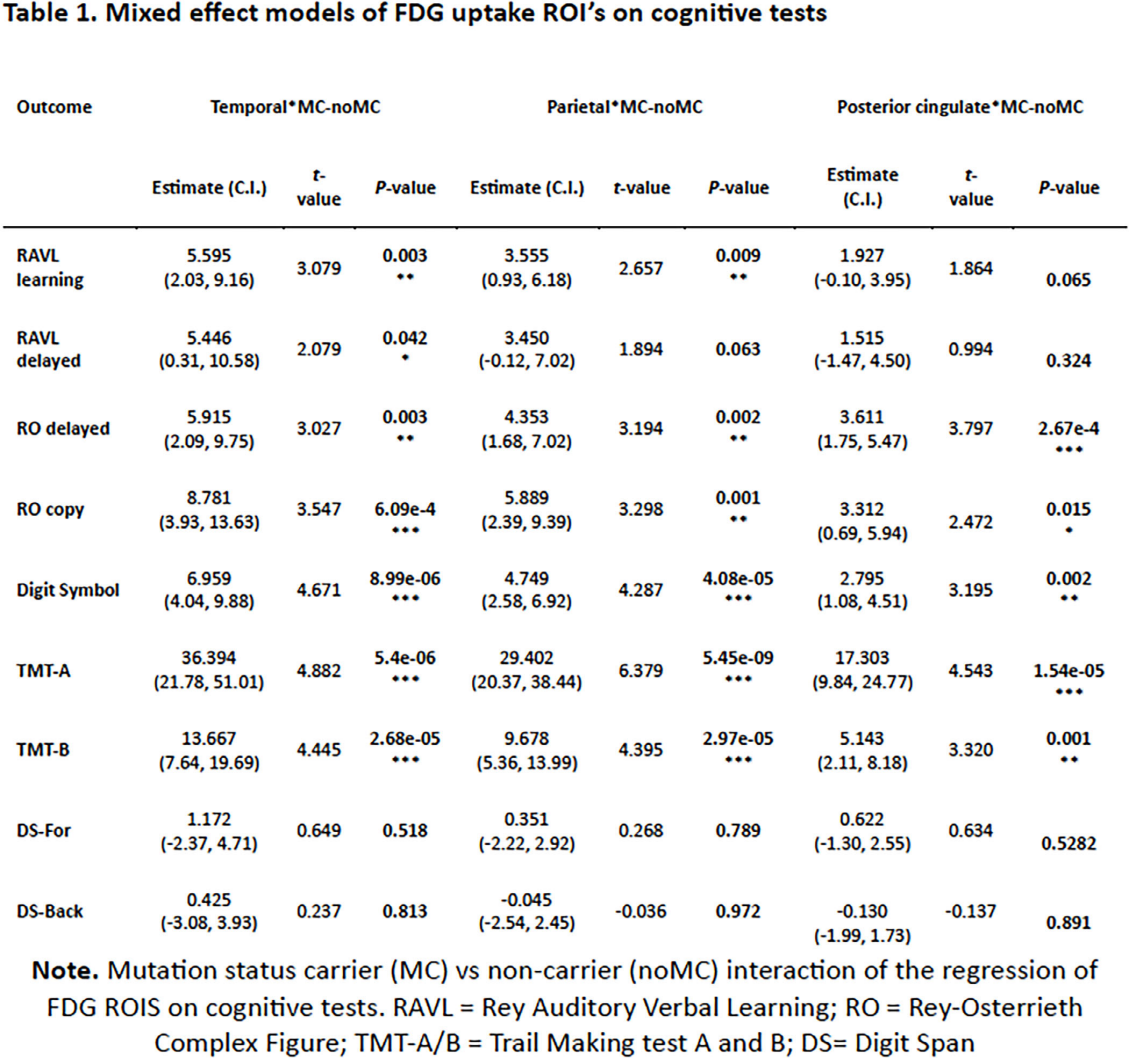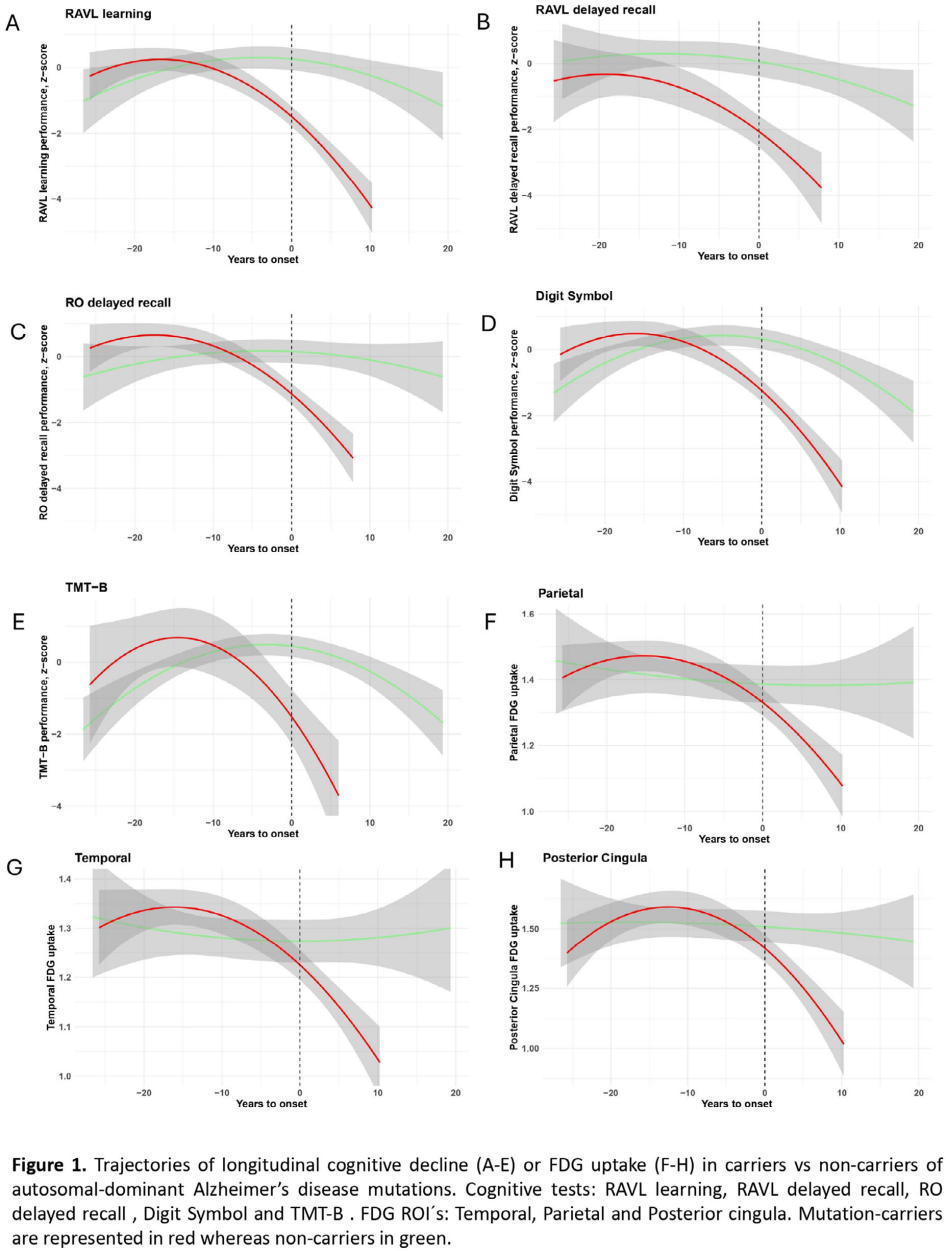# Longitudinal cognitive trajectories in ADAD: The relationship with glucose metabolism [^18^F]FDG PET

**DOI:** 10.1002/alz.086952

**Published:** 2025-01-09

**Authors:** Mariola Zapater‐Fajari, Emma S. Luckett, Konstantinos Chiotis, Marco Bucci, Anders Wall, Ove Almkvist, Elena Rodriguez‐Vieitez, Caroline Graff, Agneta K Nordberg

**Affiliations:** ^1^ Department of Neurobiology, Care Sciences and Society, Center for Alzheimer Research, Division of Clinical Geriatrics, Karolinska Institutet, Stockholm Sweden; ^2^ Karolinska Institutet, Stockholm Sweden; ^3^ Department of Neurology, Karolinska University Hospital, Stockholm Sweden; ^4^ Department of Neurobiology, Care Sciences and Society, Center for Alzheimer Research, Karolinska Institutet, Stockholm Sweden; ^5^ Turku PET Centre, Turku University Hospital, University of Turku and Åbo Akademi University, Turku Finland; ^6^ Department of Surgical Sciences, Section of Nuclear Medicine & PET, Uppsala University, Uppsala Sweden; ^7^ Theme Inflammation and Aging, Karolinska University Hospital, Stockholm Sweden; ^8^ Department of Psychology, Stockholm University, Stockholm Sweden; ^9^ Department of Neurobiology, Care Sciences and Society, Center for Alzheimer Research, Division of Neurogeriatrics, Karolinska Institutet, Stockholm Sweden; ^10^ Unit for Hereditary Dementia, Theme Inflammation and Aging, Karolinska University Hospital‐Solna, Stockholm Sweden

## Abstract

**Background:**

[^18^F]FDG PET is essential since it allows us to differentiate between different dementia disorders/types, revealing distinct neurodegenerative patterns in those predisposed to the condition. Individuals with Autosomal Dominant Alzheimer's Disease (ADAD) have a predictable age of onset, enabling the study of cognitive and pathological changes before clinical manifestation. Our objective was to investigate temporal course and regional links between cognition and glucose metabolism as a measure of early synaptic impairment in ADAD.

**Method:**

Forty‐five participants were included in the present study: 26 non‐carriers, and 19 mutation‐carriers (11 APP and 8 PSEN1) who had up to 5 measurements of [^18^F]FDG PET and neuropsychological testing within a time interval of 7.2±6.4 years. Expected years to symptom onset (EYO) in mutation‐carriers ranged from ‐25.8 to 10.3 years. Nine cognitive tests, emphasizing Episodic Memory (EM: learning and delayed recall of the Rey Auditory Verbal Learning and delayed recall of the Rey‐Ostetriech Complex Figure) and Executive Function/attention (EF/A: Digit Symbol, Rey Copy, Trail Making Test A and B, Digit Span Forward and Backward), were z‐scored using non‐mutation carriers as the reference group. Mixed‐effects models were used to identify the differential effect of mutation status on the longitudinal trajectories of cognition or [^18^F]FDG. We also assessed whether the interaction of [^18^F]FDG with mutation status could predict cognitive decline.

**Result:**

Compared to non‐carriers, all cognitive tests significantly declined across EYO in carriers (all p<0.01), except Digit Span Backward (p=0.19). Notably, some EF/A and all EM tests showed decline 15‐10 years before EYO in carriers (Figure 1A‐E). [^18^F]FDG levels started to decline in Temporal, Parietal and Posterior Cingulate regions around 10‐5 years before EYO, with significantly steeper slopes detected for carriers vs non‐carriers (all p<0.007) (Figure 1F‐H). Steeper [^18^F]FDG regional decline patterns were found to predict steeper decline over EYO in most cognitive tests in carriers compared to non‐carriers (Table 1).

**Conclusion:**

Our results link cognitive decline to [^18^F]FDG hypometabolism in several brain regions. Specific tests of EF and EM may detect early synaptic impairment due to their close relationship with [^18^F]FDG decline. This longitudinal study shed light on early changes in cognitive domains in ADAD individuals, offering insights into AD progression.